# Characterization of Trabecular Bone Microarchitecture and Mechanical Properties Using Bone Surface Curvature Distributions

**DOI:** 10.3390/jfb15080239

**Published:** 2024-08-22

**Authors:** Pengwei Xiao, Caroline Schilling, Xiaodu Wang

**Affiliations:** 1 Department of Mechanical Engineering, University of Texas at San Antonio, San Antonio, TX 78249, USA; 2 Department of Orthopedic Surgery, Massachusetts General Hospital, Harvard Medical School, 55 Fruit St., Boston, MA 02114, USA; 3 Department of Biomedical Engineering, University of Texas at San Antonio, San Antonio, TX 78249, USA

**Keywords:** surface curvature, trabecular bone, histomorphometric parameters, stiffness tensor, geometric parameter, deep learning, convolution neural network

## Abstract

Understanding bone surface curvatures is crucial for the advancement of bone material design, as these curvatures play a significant role in the mechanical behavior and functionality of bone structures. Previous studies have demonstrated that bone surface curvature distributions could be used to characterize bone geometry and have been proposed as key parameters for biomimetic microstructure design and optimization. However, understanding of how bone surface curvature distributions correlate with bone microstructure and mechanical properties remains limited. This study hypothesized that bone surface curvature distributions could be used to predict the microstructure as well as mechanical properties of trabecular bone. To test the hypothesis, a convolutional neural network (CNN) model was trained and validated to predict the histomorphometric parameters (e.g., BV/TV, BS, Tb.Th, DA, Conn.D, and SMI), geometric parameters (e.g., plate area PA, plate thickness PT, rod length RL, rod diameter RD, plate-to-plate nearest neighbor distance NND_PP_, rod-to-rod nearest neighbor distance NND_RR_, plate number PN, and rod number RN), as well as the apparent stiffness tensor of trabecular bone using various bone surface curvature distributions, including maximum principal curvature distribution, minimum principal curvature distribution, Gaussian curvature distribution, and mean curvature distribution. The results showed that the surface curvature distribution-based deep learning model achieved high fidelity in predicting the major histomorphometric parameters and geometric parameters as well as the stiffness tenor of trabecular bone, thus supporting the hypothesis of this study. The findings of this study underscore the importance of incorporating bone surface curvature analysis in the design of synthetic bone materials and implants.

## 1. Introduction

Trabecular bone, characterized by a sponge-like structure, comprises a network of plates and rods at the microstructural level [1]. The microstructure of trabecular bone is essentially important for determining bone’s resistance to fractures [2]. Currently, the major method for clinical assessment of bone microstructure is mainly based on dual-energy X-ray absorptiometry (DXA)-based trabecular bone score (TBS) [3,4], an indirect method based on bone mineral density known for its limited accuracy. Recent advancements in micro-CT technology have led to the development of micro-CT image-based reconstruction methods for evaluating trabecular bone microstructure [5,6]. This method encompasses a full set of histomorphometric parameters, including degree of anisotropy (DA), bone volume fraction (BV/TV), connectivity density (Conn.D), bone surface area (BS), structure model index (SMI), and trabecular thickness (Tb.Th), offering an overall evaluation of bone microstructure [7]. However, the histomorphometric parameters are scalar and averaged measures of trabecular microarchitecture at global levels, thus could not fully capture the variance of microarchitectural properties and their influence on mechanical properties of trabecular bone [8]. In addition, Columbia University has developed an Individual Trabeculae Segmentation ITS technique to characterize the geometric parameters of trabecular bone, allowing for the segmentation of trabecular bone into individual plates and rods [9]. Consequently, the description of bone microstructure could encompass parameters related to size (plate area PA, plate thickness PT, rod length RL, rod diameter RD), spatial arrangement (plate-to-plate nearest neighbor distance NND_PP_, rod-to-rod nearest neighbor distance NND_RR_), trabeculae number (plate number PN, rod number RN), and orientation, thus providing more microarchitectural features of bone microstructure that contribute to the mechanical competence of trabecular bone. However, these geometric parameters only provide detailed information about individual trabeculae but interpreting these parameters in the context of bone mechanical competence can be challenging. Consequently, the fundamental microarchitectural characteristics of trabecular bone remain to be fully explored. 

Recently, a novel methodology utilizing surface curvatures has been proposed for the comprehensive characterization of cancellous microstructure [10]. Various surface curvatures, such as maximum principal curvature, minimum principal curvature, Gaussian curvature, and mean curvature, offer a direct means of assessing the local geometry of bone in terms of convexity and concavity. Moreover, research has demonstrated a strong correlation between surface curvatures, SMI, and Euler number (which can be used to quantify the connectivity of trabecular bone) [11], underscoring the significance of bone surface curvatures as a pivotal metric for delineating both local and global bone geometry and effectively capturing diverse spatial structural aspects. Given the intimate relationship between bone structure and its mechanical properties, the implications of surface curvatures on bone mechanical behavior are noteworthy. Hence, understanding bone surface curvatures is crucial for bone material design as well as prediction of the mechanical behavior and functionality of bone structures. Nevertheless, to the best of our knowledge, few studies have been conducted to investigate how the surface curvatures are quantitatively related to the bone microarchitecture as well as its mechanical properties. 

In order to characterize the surface geometry of trabecular bone, it is essential to quantify bone surface curvature distributions [11]. This leads to the technical question of how to describe the surface curvature distributions by utilizing specific parameters and establishing the relationships between bone surface curvature distributions and bone microstructure. Our previous studies [12,13,14] have shown that a 2D projection image can be used to describe its 3D bone microstructure and mechanical properties by using a deep learning (DL) approach. Inspired by the aforementioned applications, we proposed to describe the curvature spatial distributions through a 2D projection image-based approach and employ a DL model to establish relationships between surface curvature distributions and bone microstructure as well as its mechanical properties.

In this study, we hypothesized that there exists a strong correlation between bone surface curvature distributions and the microstructure as well as mechanical properties of trabecular bone, and hence bone surface curvature distributions could be used as a holistic indicator for prediction of both bone microstructure and mechanical behavior. To test the hypothesis, the spatially distributed surface curvatures across the surface of trabecular bone were projected onto a two-dimensional plane, and then a deep learning model was developed to predict the histomorphometric parameters, geometric parameters, as well as mechanical properties of trabecular bone using the above two-dimensional projections of bone surface curvature distributions.

## 2. Materials and Methods

### 2.1. Preparation of Trabecular Bone Specimens and Micro-CT Image-Based Reconstruction

A total of six cadaveric proximal femurs were collected from six different donors (three males and three females, with a mean age of 48.5 ± 24 years) with Institutional Biosafety Committee (IBC) approval (IBC#B94-01-21). All proximal femurs were scanned using a micro-CT system (Sky-Scan 1173, Bruker, Billerica, MA, USA) with a resolution of 35 µm, which was able to capture the trabecular microstructure. Then, a total of eight hundred and sixty-eight trabecular cubes, each with the dimensions of 6 mm × 6 mm × 6 mm, were dissected out from the micro-CT images of the six proximal femurs to serve as representative volume elements (RVEs). It should be noted that trabecular cubes with low BV/TV and/or minimal trabeculae, as well as those containing cortical bone, were excluded from this study. Finally, all trabecular cubes were constructed digitally using STL format.

### 2.2. Calculation of Bone Surface Curvatures

**Figure 1:**
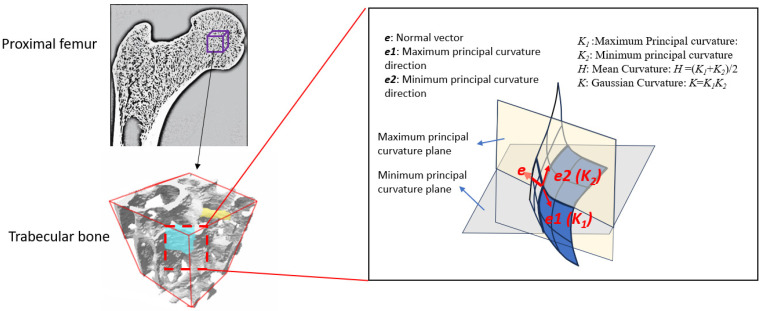
The schematic represents trabecular bone surface curvature using maximum principal curvature (*K*_1_), minimum principal curvature (*K*_2_), mean curvature (*H*), and Gaussian curvature (*K*).

Surface curvatures of trabecular bone were computed utilizing the STL format of the reconstruction of trabecular bone. A previous study demonstrated that bone surface curvatures, including the principal curvatures, mean curvatures, and Gaussian curvatures, could distinguish bone microstructure across different locations, suggesting their potential for predicting bone failure [10]. Therefore, the maximum and minimum principal curvatures, as well as the mean and Gaussian curvatures (Figure 1), were employed to define the surface curvature of the trabecular bone in this study, as these surface curvatures could capture the most fundamental shape details [10]. Specifically, the maximum and minimum principal curvatures (*K*_1_, *K*_2_) of the trabecular bone surface were computed on the triangle meshes of the trabecular bone surface using a finite-differences approach [15]. Subsequently, Gaussian curvature *K* and mean curvature *H* were defined as follows:

*K* = *K*_1_*K*_2_ (1)

*H* = (*K*_1_ + *K*_2_)/2 (2)

The above four types of surface curvatures were computed for each vertex of the triangle meshes in each trabecular cube using MATLAB R2023a (The MathWorks, Inc., Natick, MA, USA).

### 2.3. Characteristics of Trabecular Microarchitecture

To describe the microarchitecture of trabecular bone globally, six histomorphometric parameters were measured from the micro-CT images of the trabecular bone cubes using ImageJ (1.52 h) and BoneJ (https://bonej.org/). These six histomorphometric parameters included bone volume fraction (BV/TV), bone surface area (BS), trabecular thickness (Tb.Th), structure model index (SMI), the degree of anisotropy (DA), and connectivity density (Conn.D). Using these six histomorphmetric parameters, trabecular bone mass, trabecular size, number, structure types (either plate-like or rod-like), and trabecular orientation can be defined accurately.

Moreover, the geometric parameters of trabecular bone cubes were also assessed utilizing a novel individual trabecula segmentation (ITS) technique [9]. With recent advancements in biomedical image processing technologies, the microarchitecture of trabecular bone can be segmented into individual trabecular plates and rods, allowing describing the microarchitecture of trabecular bone using trabecular number (plate number PN, rod number RN), trabecular size (mean plate thickness PT, mean plate area PA, mean rod diameter RD, mean rod length RL), trabecular arrangement (mean plate-to-plate nearest neighbor distance NND_PP_, mean rod-to-rod nearest neighbor distance NND_RR_), and trabecular orientation [8,16]. Using the above parameters, the geometry of trabecular bone could be precisely defined.

### 2.4. Determination of the Mechanical Properties of Trabecular Bone Using the Micro-FE Method

The anisotropic mechanical behavior of trabecular cubes can be described in terms of the apparent stiffness tensor. The stiffness tensor of trabecular cubes, which is a fourth-rank tensor and is considered elastically orthotropic with three mutually perpendicular planes of symmetry [17,18], can be simplified as

(3) $$C=\left[\begin{array}{cc} \begin{array}{ccc} C_{11} & C_{12} & C_{13}\\ C_{21} & C_{22} & C_{23}\\ C_{31} & C_{32} & C_{33} \end{array} & \begin{array}{ccc} 0 & 0 & 0\\ 0 & 0 & 0\\ 0 & 0 & 0 \end{array}\\ \begin{array}{ccc} 0 & 0 & 0\\ 0 & 0 & 0\\ 0 & 0 & 0 \end{array} & \begin{array}{ccc} C_{44} & 0 & 0\\ 0 & C_{55} & 0\\ 0 & 0 & C_{66} \end{array} \end{array}\right]$$

where *C*_12_ = *C*_21_, *C*_13_ = *C*_31_, and *C*_23_ = *C*_32_. In this study, trabecular cubes with the dimension of 6 mm × 6 mm × 6 mm dissected out from the various anatomic regions of the femurs, such as the femur head, neck, and greater trochanter regions, were used as RVEs, and the stiffness tensor of trabecular cubes was assessed using micro-CT-based finite element (FE) simulations. The FE analysis was conducted using Abaqus 2021/Standard software package. Specifically, a direct voxel conversion method was employed to transform each voxel of the digitized trabecular cubes into first-order tetrahedral elements (C3D4), generating approximately 0.5 to 2.5 million tetrahedral elements for each trabecular cube. Trabecular bone was assumed to be homogeneous, linearly elastic, and isotropic material, with a Young’s modulus of 15 GPa and a Poisson’s ratio of 0.3 [19]. Then, six uniform boundary conditions, including three uniaxial compression tests along the three orthogonal coordinate axes and three pure shear tests in the three orthogonal planes, were applied to the FE model sequentially to assess the stiffness matrix. Finally, the stiffness tensor was obtained by rotating the fabric coordinate axes of the stiffness matrix to its principal axes [20] using the MSAT (a toolkit for the analysis of elastic and seismic anisotropy) in a MATLAB environment.

### 2.5. Development of DL Model

#### 2.5.1. Characterization of Bone Surface Curvature Distributions Using a 2D Projection Image-Based Approach

**Figure 2:**
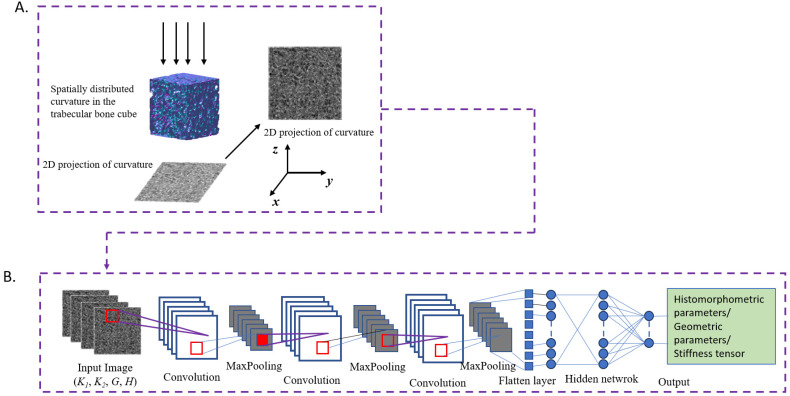
The schematic framework DL model based on 2D projections of trabecular surface curvatures (maximum principal curvature (*K*_1_), minimum principal curvature (*K*_2_), mean curvature (*H*), and Gaussian curvature (*K*)). (**A**). Projection of trabecular surface curvatures onto a 2D plane. (**B**). The architecture of the CNN model with the 2D projection images of curvatures as input and the histomorphometric parameters/geometric parameters/stiffness tensor as output.th.

In this study, the spatial distributions of bone surface curvatures on each vertex of the triangle mesh of trabecular bone were characterized by projecting the surface curvatures onto a 2D plane (Figure 2). Our previous studies have shown that the 2D projection of properties effectively captures their 3D spatial distribution and could be effectively learned by the DL model. Thus, we projected the bone surface curvatures, including maximum principal curvature, minimum principal curvature, Gaussian curvature, and mean curvature, onto four different 2D planes for each trabecular cube using custom MATLAB scripts (MathWorks, Natick, MA, USA). Briefly, the 2D plane was meshed at the resolution of 172 pixels (bins) × 172 pixels (bins), matching the resolution of the micro-CT images for each trabecular cube. Next, the trabecular cubes were meshed along the projection direction with a thickness of one voxel, generating n = 172 planer layers. Then, the curvature values at each vertex in each planer layer of the trabecular cubes were projected to the 2D plane. The curvature values at each bin were obtained by summing all the curvature values falling onto the bin using the following equation:

(4) $$K\left(x,y\right)=\frac{1}{n}\sum_{z=1}^{n}k(x,y,z)$$

where, *K* is the curvature value of the bin at the location (*x*, *y*) on the 2D projection plane; *k* is the summation of the curvature values at the location (*x*, *y*, *z*) in the trabecular cube; *n* is the number of plane layers of the trabecular cube in the projection direction (*n* = 172). Finally, the 2D projection plane was converted into a 2D image. In this study, the 2D projection images of maximum principal curvature, minimum principal curvature, Gaussian curvature, and mean curvature were used as input to train the DL model.

#### 2.5.2. Convolutional Neural Network (CNN) Modeling

In order to explore the correlations between bone surface curvature distributions and the microstructure and mechanical properties of trabecular bone, one CNN model was developed and trained in this study to predict the histomorphometric parameters, geometric parameters, as well as mechanical properties of trabecular bone based on the 2D projection images of bone surface curvatures. The architecture of the proposed CNN model is illustrated in Figure 2, consisting of multiple convolutional layers, max-pooling layers, and a fully connected neural network followed by the outputs. During the training process, the 2D projection images of bone surface curvatures were used as input, while the histomorphometric parameters, geometric parameters, and the apparent stiffness tensor were used as output, respectively. The mean square error (MSE) was utilized as a loss function throughout the training process. Furthermore, hyperparameter optimization was conducted to meticulously refine the architecture of the CNN model to achieve optimal performance across the training process. The parameters assessed in the CNN architecture comprised the number of hidden layers, the number of filters, the number of convolutional layers, kernel size, the optimizer functions, the learning rates, the number of epochs, and the dropout rate. Finally, the details of the optimized architecture of the CNN model were shown in Table 1.

In addition, in order to minimize the effect of different scales on the results, all output parameters were normalized by using rescaling (min-max normalization) before training the CNN model using the following formula:

(5) $$x^{\prime }=\frac{x-\min\;(x)}{\max\left(x\right)-\min\;(x)}$$

where *x* is the original value, *x*′ is the normalized value.

In this study, 80% of the datasets were randomly selected as training datasets, while the remaining 20% were used as testing datasets. The CNN model was programmed in Python using the Keras library with a TensorFlow backend and was trained on a Dell desktop computer (XPS 8930, Intel Core i9-9900k 8-Core Processor, 64 GB Memory, NVIDIA R GeForce^®^ GTX 1080 with 8 GB GDDR5X Graphic Memory, Dell, Round Rock, TX, USA).

### 2.6. Data Analysis

The correlations between the distributions of bone surface curvature and the histomorphometric parameters, geometric parameters, as well as the mechanical properties of trabecular bone were evaluated by quantifying the prediction accuracy of the DL model in predicting those parameters. By performing the linear regression analyses, the prediction accuracy of the DL model was assessed using the Pearson correlation coefficient (*R*^2^), with significance determined at *p* < 0.05. All the statistical analyses were performed using IBM SPSS software (version 29, IBM, Chicago, IL, USA).

## 3. Results

### 3.1. Correlation between Bone Aurface Curvature Distributions and Histmorphometric Parameters of Trabecular Bone

**Figure 3:**
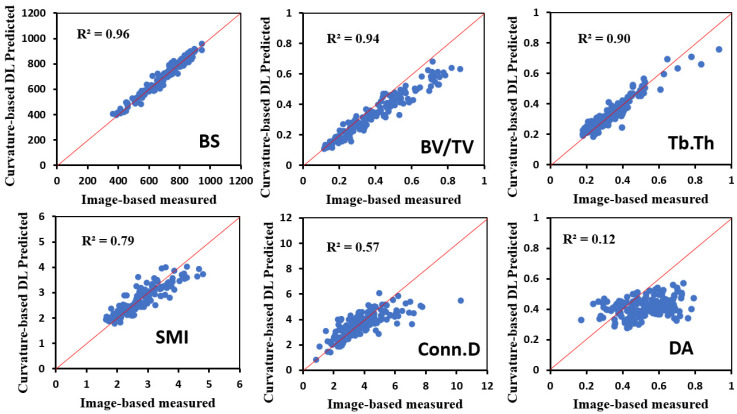
Regression plots of the microstructural parameters predicted by the curvature-based DL model vs. measured by micro-CT images.

The linear regression analyses were performed to assess the prediction accuracy of the surface curvature-based DL model in predicting the histomorphometric parameters (Figure 3). The results showed that the histomorphometric parameters predicted by the surface curvature-based DL model were consistent with those measured directly from micro-CT images. The Pearson correlation coefficients (*R*^2^) were 0.96, 0.94, 0.90, 0.79, 0.57, and 0.11 for bone surface (BS), bone volume fraction (BV/TV), trabecular thickness (Tb.Th), structural model index (SMI), connectivity density (Conn.D), and the degree of anisotropy (DA), respectively, with all the *p*-values < 0.0001. Employing *R*^2^ as an indicator of predictive accuracy of the surface curvature-based DL model, the results suggest that bone surface curvature distributions were significantly correlated with BS, BV/TV, Tb.Th, SMI, and Conn.D, with the exception of DA. Moreover, bone surface curvature distributions exhibited the highest Pearson correlation coefficient with BS among the histomorphometric parameters, whereas a weak correlation was observed between bone surface curvature distributions and DA.

### 3.2. Correlation between Bone Surface Curvature Distributions and Geometric Parameters of Trabecular Bone

**Figure 4:**
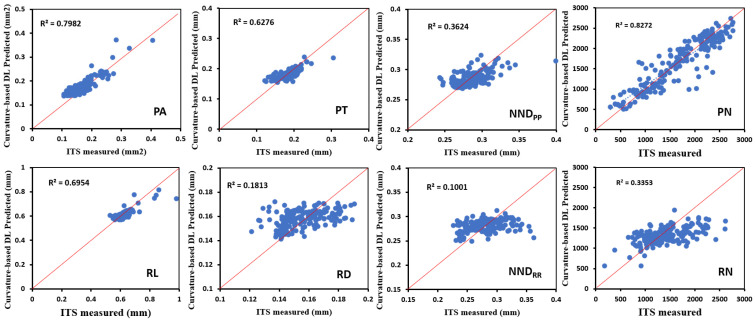
Regression plots of the geometric parameters predicted by the curvature-based DL model vs. measured by micro-CT images.

The linear regression analyses were also used to assess the prediction accuracy of the geometric parameters, including trabecular size, spatial arrangement, and trabecular number (Figure 4). The results indicated the surface curvature-based DL model exhibited reasonably high accuracy in predicting plate area (PA), plate thickness (PT), and rod length (RL), with Pearson correlation coefficients *R*^2^ of 0.80, 0.63, and 0.79, respectively. However, the model demonstrated lower prediction accuracy for rod diameter (RD), plate-to-plate nearest neighbor distance (NND_PP_), and rod-to-rod nearest neighbor distance (NND_RR_), with R^2^ values of 0.36, 0.36, and 0.10, respectively. These findings indicated strong correlations between bone surface curvature distributions and PA, PT, and RL but weak correlations with RD, NND_PP_, and NND_RR_. Additionally, the Pearson correlation coefficients *R*^2^ were 0.83 for plate number PN and 0.34 for rod number RN, suggesting a strong correlation between bone surface curvature distributions and plate number but a weak correlation with rod number.

### 3.3. Correlation between Bone Surface Curvature Distributions and Apparent Stiffness Tensor of Trabecular Bone

**Figure 5:**
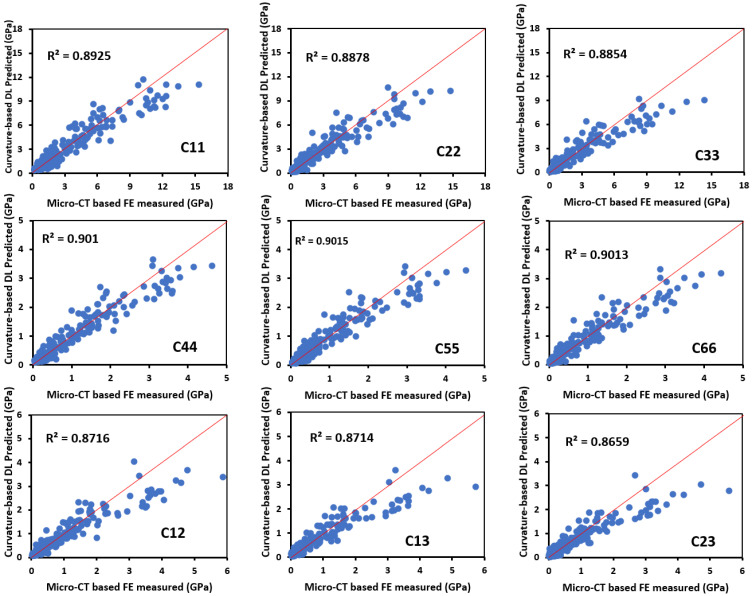
Regression plots of the stiffness tensor predicted by the curvature-based DL model vs. measured by micro-CT images.

The prediction accuracy of the bone surface curvature-based DL model in predicting the constant components of the apparent stiffness tensor was assessed by comparing the DL-predicted stiffness tensor with the ground-true values measured using FE simulations. The Pearson correlation coefficients (*R*^2^) were 0.89, 0.89, 0.88, 0.90, 0.90, 0.90, 0.87, 0.87, and 0.86 for the apparent stiffness tensor constants *C*_11_, *C*_22_, *C*_33_, *C*_44_, *C*_55_, *C*_66_, *C*_12_, *C*_13_, and *C*_23_, respectively, with all the *p*-values < 0.001 (Figure 5), suggesting high correlations between bone surface curvature distributions and the apparent stiffness tenor of trabecular cube. These findings imply that bone surface curvature distributions could be used to predict the anisotropic mechanical behavior of trabecular bone with high accuracy.

## 4. Discussion

This study investigated the correlations between bone surface curvature distributions and trabecular microstructure as well as mechanical properties using a DL approach. A surface curvature-based CNN model was developed and trained to predict the histomorphometric parameters, geometric parameters, and the apparent stiffness tensor of trabecular bone. The results demonstrated that bone surface curvature distributions were not only highly correlated with the major histomorphometric parameters and geometric parameters, but also with the apparent stiffness tensor of trabecular bone. These findings supported the hypothesis that bone surface curvature distributions can serve as a holistic parameter for predicting bone microstructure and mechanical behavior with reasonably high accuracy, underscoring the significance of incorporating bone surface curvature analysis in the design of synthetic bone materials and implants.

**Figure 6:**
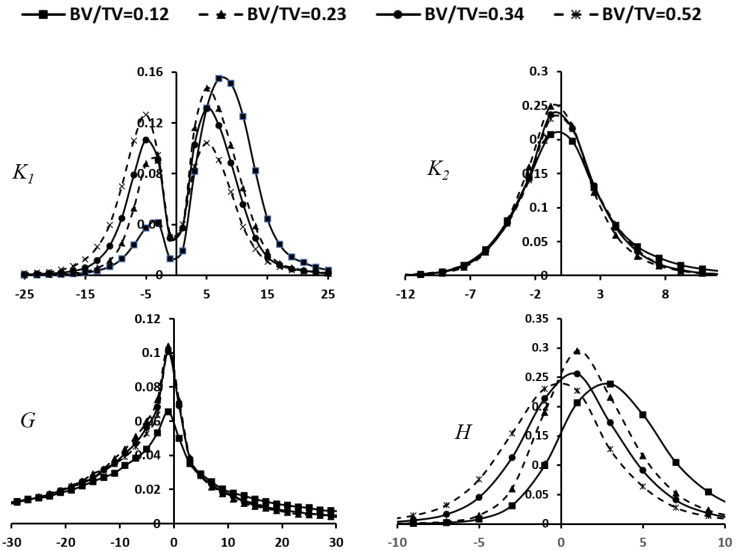
Probability distribution of surface curvatures (maximum principal curvature *K*_1_, minimum principal curvature *K*_2_, Gaussian curvature *K*, and mean curvature *H*) vs. bone volume fraction (BV/TV).

Previous studies have indicated that bone surface curvatures primarily capture the local geometry of trabecular bone [11]. However, our study reveals that bone surface curvature distributions could be used to effectively predict the histomorphometric parameters of trabecular bone using the DL model, suggesting that bone surface curvature distributions can be used to evaluate the overall changes of bone microstructure. The results in this study demonstrated bone surface curvature distributions exhibited the strongest correlation coefficients with bone BS (*R*^2^ = 0.96) and Tb.Th (*R*^2^ = 0.90) among the histomorphometric parameters. Previous studies have shown that BS is a function of the integration of bone surface curvature [11]. Given the strong correlation between bone surface curvatures and BS as well as Tb.Th, it is reasonable to infer a similarly strong correlation with bone volume fraction (BV/TV) (*R*^2^ = 0.94). Indeed, by plotting the distributions of bone curvature of trabecular cubes with different BV/TV values (Figure 6), BV/TV can be clearly identified by the distributions of bone surface curvatures. In addition, serval studies have demonstrated the correlation between bone surface curvatures and SMI [11]. This study is the first attempt to predict SMI using bone surface curvature distributions with high accuracy (*R*^2^ = 0.79). Furthermore, this study also finds a reasonable correlation between bone surface curvature distributions and Conn.D. but a low correlation between bone surface curvatures and DA. Nonetheless, this study demonstrated bone surface curvature distributions could accurately predict the major histomorphometric parameters of trabecular bone with reasonable accuracy, suggesting the significance of bone surface curvature distributions in assessing the overall microstructural changes of trabecular bone.

This study also investigated the correlations between bone surface curvature distributions and the geometric parameters measured using individual trabecula segmentation (ITS) analysis, such as trabecular size, spatial arrangement, and trabecular number. These parameters describe the local microstructural features of trabecular bone as well as its mechanical properties [8]. High correlations were found between bone surface curvature distributions and parameters such as PA, PT, and RL, with *R*^2^ values of 0.80, 0.63, and 0.70, respectively, suggesting that bone surface curvature distributions are sensitive to the changes in trabecular plate area, plate thickness, and rod length. However, a low correlation was observed between bone surface curvature distributions and rod diameter (RD) (*R*^2^ = 0.18), implying that bone surface curvature distributions might not be able to accurately capture the changes in rod diameter. In addition, it is interesting to find that there were strong correlations between bone surface curvature distributions and plate number (R^2^ = 0.83), an important parameter closely related to the onset of osteoporosis. It has been observed that plate-like trabeculae were seriously depleted in patients with osteoporotic fractures [21]. The results in this study further demonstrated that bone surface curvature distributions are able to pick up the local changes in bone microstructure during the onset of osteoporosis.

To the best of our knowledge, this study represents the first attempt to predict the mechanical properties of trabecular bone using a DL model based on the distributions of bone surface curvatures. The strong correlations between bone surface curvature distributions and stiffness tensors of trabecular bone (*R*^2^ = 0.89–0.91) demonstrated the capability of surface curvature distributions in predicting the anisotropic mechanical properties of trabecular bone. This suggests that bone surface curvature distributions can serve as a novel parameter for governing the mechanical behavior of trabecular bone. Previous studies have shown that the structure-function of trabecular bone is mainly attributed to a full set of histomorphometric parameters [14,22], such as BV/TV, SMI, Conn.D, Tb.Th, BS, and DA. However, the changes in individual histomorphometric parameters might not be able to fully reflect the changes in mechanical behavior. Instead of using a full set of histomorphometric parameters, the high correlations between bone surface curvature distributions and the microstructure as well as mechanical properties allow bone surface curvature distributions to be a holistic parameter in capturing the subtle changes in bone microstructure as well as bone mechanical properties.

**Figure 7:**
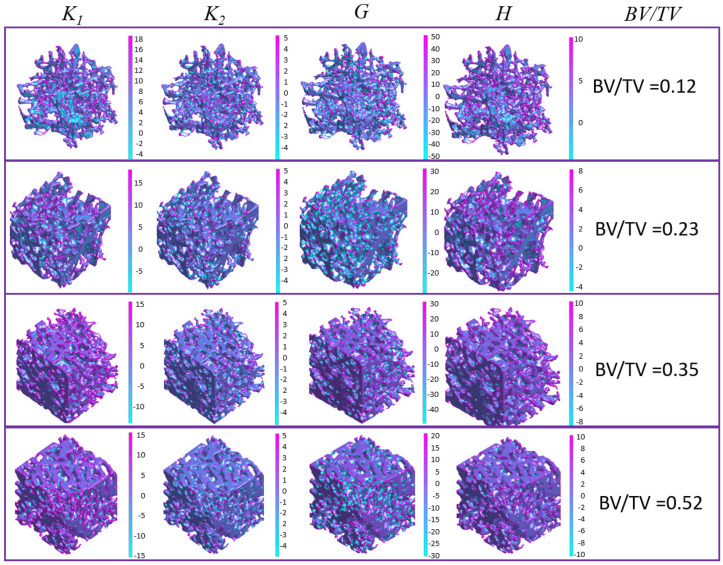
Plots of surface curvatures (maximum principal curvature *K*_1_, minimum principal curvature *K*_2_, Gaussian curvature *K*, and mean curvature *H*) over trabecular surface.

Indeed, the spatial distributions of bone surface curvatures could reveal various geometric characteristics of bone microstructures (Figure 7). By examining the spatial distributions of bone surface curvatures, the maximum principal curvature and mean curvature seem to effectively characterize the overall framework of bone microstructure, while the minimum principal curvature and Gaussian curvature are more appropriate for capturing local topological characteristics. Moreover, previous studies have shown that mean curvature describes the local convexity or concavity of a surface, whereas Gaussian curvature delineates different surface types, including saddle-shaped regions (*K* < 0), intrinsically flat regions (*K* = 0), and sphere-shaped regions (*K* > 0) [10]. Additionally, this study investigated the correlations between individual surface curvature distributions and the histomorphometric parameters of trabecular bone using DL models. The results (Table 2) showed that DL models based on maximum principal curvature distribution and mean curvature distribution demonstrated higher prediction accuracy in predicting bone surface area, whereas Gaussian curvature-based DL models demonstrated higher prediction accuracy in predicting trabecular thickness, thus suggesting that maximum principal curvature and mean curvature are more closely correlated with bone surface area while Gaussian curvature is more strongly correlated with trabecular thickness. Furthermore, the results indicated that the prediction accuracy of DL models using individual surface curvature as input is comparable to that of DL models using all four surface curvatures as input, implying that each surface curvature distribution contains the major geometric characteristics regarding bone microstructure. 

Moreover, surface curvatures have been extensively applied in various fields. Several studies have been conducted on applying bone surface curvatures for segmenting and labeling bone surface regions due to their reliable detection of geometric features [23,24,25,26]. Furthermore, researchers have applied bone surface curvature to fabricate tissue scaffolds [27], indicating that bone surface curvature allows to create a library of mathematically designed scaffolds, showing its potential for regeneration of anisotropic bone structure. Guo et al., proposed a deep learning approach for designing structures with targeted surface curvature [28], which could be designed to promote mechanical behavior. Researchers further examined the effects of the curvature of the femur and tibia on biomechanical behavior during unloaded uphill locomotion [29], highlighting the strong correlation between bone curvature and locomotor function, as well as underlying skeletal structure. These findings demonstrated the significant role of bone surface curvature in governing the structure and mechanical behavior of bone.

Several limitations should be acknowledged in this study. Firstly, training a robust DL model typically necessitates a comprehensive dataset. However, this study included only six proximal femurs from six distinct donors, which might not represent the general population’s bone surface curvatures. Nonetheless, the findings of this study are still valid to support the hypothesis of this study. Secondly, the projection of bone surface curvature distributions onto a 2D plane might not fully capture the spatial distributions of surface curvature by DL model. Future studies could investigate the prediction of bone microstructure as well as mechanical behavior using 3D surface curvature distributions as input for the DL model.

## 5. Conclusions

This study is the first to quantitatively correlate bone surface curvature distributions with both the microstructure and the mechanical behavior of trabecular bone using a deep learning (DL) model, demonstrating that bone surface curvature distributions can serve as a novel parameter governing the microstructure and mechanical behavior of trabecular bone. The DL model based on the surface curvature distributions demonstrated a high fidelity in predicting the microstructure as well as the mechanical properties of trabecular bone, thus verifying the hypothesis of this study. In addition, the following conclusions could be achieved: Firstly, the maximum principal curvature and mean curvature could effectively capture the overall framework of bone microstructure, whereas the minimum principal curvature and Gaussian curvature are better suited for capturing the local topological features. Secondly, each surface curvature distribution contains the major geometric characteristics regarding bone microstructure. Finally, bone surface curvature could serve as a holistic parameter in describing the bone microstructure and mechanical behavior. The findings of this study underscore the significance of incorporating bone surface curvature analysis in the design of synthetic bone materials and implants.

## Figures and Tables

**Table 1:** Optimized architecture of CNN model in prediction of histomorphometric parameters, geometric parameters, and stiffness tensor using bone surface curvature distributions.

Models	Input	Kernel Size	Pool Size	Convolutional Layers	# of Hidden Layers	Learning Rates	No. of Epochs	No. of Filters	Drop-Out	Output
#1	Bone surface curvature distributions	3 × 3	2 × 2	(8, 16, 32)	3	0.0001	200	128 × 64 × 6	0.3	Histomorphometric parameters
#2		5 × 5	2 × 2	(16, 16, 64)	3	0.0001	300	128 × 64 × 8	0.4	Geometric parameters
#3		3 × 3	2 × 2	(16, 32, 64)	3	0.0001	250	128 × 64 × 9	0.5	Stiffness tensor

**Table 2:** Comparison of prediction accuracies of microstructural parameters of trabecular bone using different inputs for DL model.

Inputs for DL Model	Prediction Accuracy (*R*^2^)					
	BS	BV/TV	Tb.Th	SMI	Conn.D	DA
*K*_1_	0.94	0.92	0.86	0.77	0.42	0.11
*K*_2_	0.91	0.91	0.85	0.76	0.44	0.11
*G*	0.84	0.92	0.91	0.77	0.37	0.08
*H*	0.94	0.91	0.88	0.76	0.47	0.06
*K*_1_, *K*_2_, *G*, *H*	0.96	0.94	0.90	0.79	0.57	0.12

## Data Availability

The original contributions presented in the study are included in the article, further inquiries can be directed to the corresponding author.
